# Enhanced Colorimetric Signal for Accurate Signal Detection in Paper-Based Biosensors

**DOI:** 10.3390/diagnostics10010028

**Published:** 2020-01-07

**Authors:** Dorin Harpaz, Evgeni Eltzov, Timothy S. E. Ng, Robert S. Marks, Alfred I. Y. Tok

**Affiliations:** 1 School of Material Science & Engineering, Nanyang Technology University, 50 Nanyang Avenue, Singapore 639798, Singapore; DORIN001@e.ntu.edu.sg (D.H.); sientimo001@e.ntu.edu.sg (T.S.E.N.); 2 Department of Biotechnology Engineering, Ben-Gurion University of the Negev, Beer-Sheva 84105, Israel; 3 Institute for Sports Research (ISR), Nanyang Technology University and Loughborough University, Nanyang Avenue, Singapore 639798, Singapore; 4 Agriculture Research Organization (ARO), Volcani Centre, Rishon LeTsiyon 15159, Israel; eltzov@volcani.agri.gov.il; 5 The National Institute for Biotechnology in the Negev, Ben-Gurion University of the Negev, Beer-Sheva 84105, Israel; 6 The Ilse Katz Centre for Meso and Nanoscale Science and Technology, Ben-Gurion University of the Negev, Beer-Sheva 84105, Israel

**Keywords:** colorimetric signal, paper-based biosensors, point-of-care, enzyme horseradish peroxidase (HRP), 3,3’,5,5’-tetramethylbenzidine (TMB), 2’-azinobis (3 ethylbenzothiazoline-6-sulfonic acid) (ABTS)

## Abstract

Paper-based colorimetric biosensors combine the use of paper with colorimetric signal detection. However, they usually demonstrate lower sensitivities because a signal amplification procedure has not been used. Stopping the reaction of colorimetric signal generation is often used in lab-based assays in order to amplify and stabilize the colorimetric signal for detection. In this study, the generation of a stopped colorimetric signal was examined for accurate and enhanced signal detection in paper-based biosensors. The colorimetric reaction in biosensors is usually based on the interaction between the enzyme horseradish peroxidase (HRP) and a selected chromogenic substrate. The two most commonly used HRP substrates, 3,3’,5,5’-tetramethylbenzidine (TMB) and 2’-azinobis (3-ethylbenzothiazoline-6-sulfonic-acid) (ABTS), were compared in terms of their ability to generate a stopped colorimetric signal on membrane. The stopped colorimetric signal was visible for TMB but not for ABTS. Moreover, the generation of stopped colorimetric signal was dependent on the presence of polyvinylidene-difluoride (PVDF) membrane as the separation layer. With PVDF the colorimetric signal (color intensity) was higher (TMB: 126 ± 6 and ABTS: 121 ± 9) in comparison to without PVDF (TMB: 110 ± 2 and ABTS: 102 ± 4). The TMB stopped colorimetric signal demonstrated a more stable signal detection with lower standard deviation values. To conclude, a stopped colorimetric signal can be generated in paper-based biosensors for enhanced and accurate signal detection.

## 1. Introduction

The three main advantages of biosensors include simplicity, cost-effectiveness and rapid results. Colorimetric detection puts to best use these important biosensors advantages. The current technologies that are based on colorimetric detection are mainly focused on point-of-care platforms, miniaturization of size, reduction of cost and without the incorporation of additional instruments [1,2,3]. A colorimetric sensor is based on the detection of analytes via a change in color that can be observed visually. Colorimetric sensors are categorized according to the different molecular interaction. Chemical or biomolecular-based interactions are categorized as chemical-sensors or biosensors respectively. Biosensors allow the detection of proteins, amino acids, nucleic acids, bacteria and pathogens. Whereas, chemical-sensors mainly detect organic compounds, heavy-metals, toxic gases and explosives [4,5,6]. Paper-based colorimetric biosensors combine the use of paper diagnostics with colorimetric signal detection. They are attractive due to their simple fabrication, accessibility, and low-cost [7]. The use of paper for biosensor technologies show two main advantages, which are sample capillary flow and compatibility with biomolecules [8]. Although, they still exhibit lower sensitivity and accuracy [9,10]. Paper-based colorimetric biosensors often exhibit low sensitivities because a signal amplification procedure was not used. Therefore, the current research is focused on signal amplification procedures for enzyme-mediated reactions [11].

Colorimetric biosensing main challenge is to transform the biomolecule detection event into a reaction of a visible change in color. The colorimetric reaction in paper-based biosensors is mainly based on the interaction between the labelled antibody–protein immunocomplex and a selected chemical substrate. Most commercially available antibodies are labelled with the enzyme horseradish peroxidase (HRP), and are used in immunoassay developments [12,13,14]. The traditional enzyme-linked immunosorbent assay (ELISA) show the use of an HRP-labelled secondary antibody. The secondary antibody is used in order to quantify the binding reaction between the target analyte and the specific primary antibody. This specific binding interaction is then detected by measuring the oxidizing reaction of HRP enzyme with a chromogenic substrate [15]. The oxidizing reaction occurs in the presence of hydrogen peroxide that is the natural substrate. The HRP enzyme breaks two hydrogen peroxide molecules into water and oxygen. However, the specificity of the HRP enzyme for the second molecule of hydrogen peroxide is low and therefore other electron donors may be considered. This low specificity increased the development of additional chromogenic substrates for HRP enzyme. The hydrogen donors substrates are oxidized and form a colored product that can be spectrophotometrically monitored [12]. There are several well studied HRP chromogenic substrates, such as: 3,3’,5,5’-tetramethylbenzidine (TMB); 2’-azinobis (3-ethylbenzothiazoline-6-sulfonic acid) (ABTS); o-phenylenediamine (OPD); 5-aminosalicylic acid (5-AS); 3-amino-9-ethylcarbazole (AEC); 3-methyl-2-benzothiazolinone hydrazone (MBTH); 3,3’-diaminobenzidine (DAB) and 4-chloro-1-naphthol (4-CN) [16,17,18]. In addition, the detection of the colorimetric signal can be further enhanced, in order to allow a more accurate signal measurement using a selected stopping solution [19,20].

**Figure 1:**
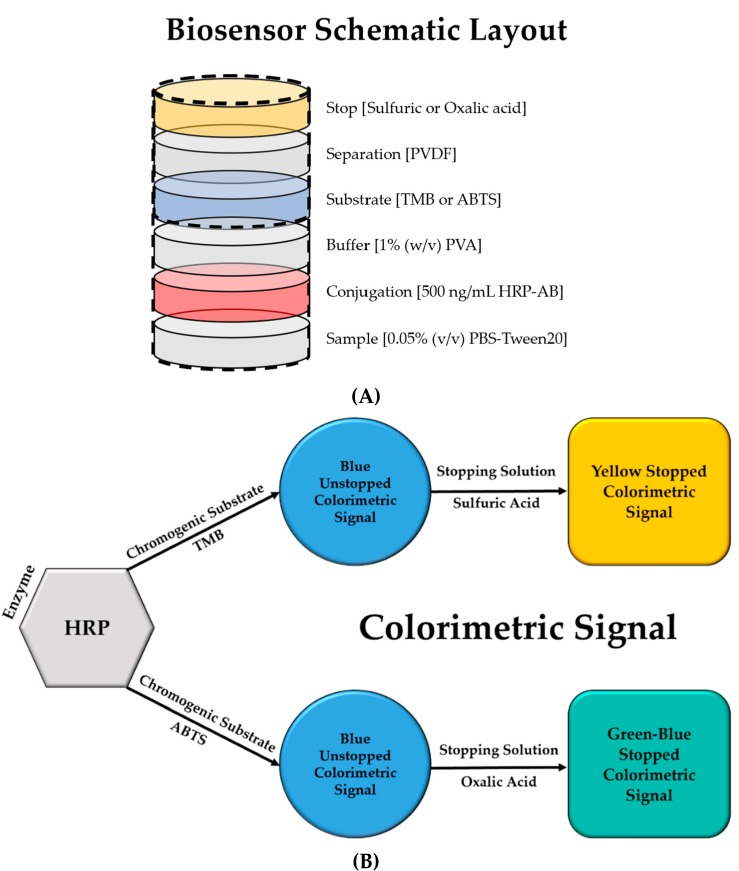
Biosensor schematic description. (**A**) The detection of stopped colorimetric signal on membrane was examined using ‘Stack-Pad’ sensor layout [21,22]. (**B**) The colorimetric signal was generated based on the reaction of the enzyme horseradish peroxidase (HRP) with the chromogenic substrates 3,3’,5,5’-tetramethylbenzidine (TMB) and 2’-azinobis (3-ethylbenzothiazoline-6-sulfonic acid) (ABTS). Sulfuric acid and oxalic acid were used as the stopping solutions for TMB and ABTS respectively.

In this study, the generation of a stopped colorimetric signal was examined for an accurate and enhanced signal detection in paper-based biosensors. Stopping the reaction of colorimetric signal generation not only enhances the signal, it also stabilizes it in order to allow a more accurate signal detection. The two most commonly used HRP substrates, TMB and ABTS, were compared in terms of their ability to generate a stopped colorimetric signal on membrane. First, the stopped colorimetric signals were compared in solution. Three different concentrations of stopping solutions were tested for each substrate. Later, for a more accurate comparison between unstopped and stopped colorimetric signals detection in paper-based biosensors, the unstopped colorimetric signal on membrane was also examined. Subsequently, the stopped colorimetric signals were detected on membranes for paper-based biosensors, using a ‘Stack-Pad’ sensor layout [21,22,23], which consists of vertically stacked functional membranes (Figure 1). The unstopped colorimetric signal was detected with and without the use of polyvinylidene difluoride (PVDF) [24] as a separation layer between the two functionalized layers of cellulose absorption pads, containing substrate and stop solution [25].

## 2. Materials and Methods

### 2.1. Materials

Phosphate buffered saline (PBS) tablets (Cat. No. P4417), polyoxyethylene sorbitan monolaurate (Tween20) (Cat. P7949), skim Milk powder (Cat. 70166), TMB liquid substrate for ELISA (Cat. No. T0440), sulfuric acid (Cat. No. 84716) and oxalic acid (Cat. No. 241172) were purchased from Sigma-Aldrich. Glassfiber (GFB-R4), polyester (PT-R5) and cellulose (AP080) were purchased from MDI membrane technologies. Nitrocellulose pore size 0.45 µm (1620115) and PVDF pore size 0.2 µm (1620177) were purchased from Bio-Rad. HRP Conjugated goat anti human IgG (Cat. ab97175) was purchased from Abcam. Recombinant protein G (Cat. 21193) and ABTS single solution ready for use (Cat. No. 00-2024) were purchased from Thermo Fisher. Milli-Q ultrafiltered (UF) H_2_O (with a resistivity of 18.2 MΩ cm at 25 °C) were used in the preparation of all solutions.

### 2.2. Equipment

Weighing balance was purchased from Mettler Toledo. The hotplate (WH220PLUS) and the Binder oven were purchased from Gaia Science Pte Ltd. Membrane cutter (PAT/RE38, 219 and D613, 795) was purchased from EK Tools, Vaessen Creative (Vaessen B.V. Thermiekstraat 25 6361HB Nuth, The Netherlands). Digital Vernier Caliper was purchased from Kincrome. Luminoskan Ascent Luminometer was purchased from Thermo Fisher Scientific (Waltham, MA, USA). TECAN Infinite M200 PRO was purchased from Tecan Trading AG, Switzerland (Seestrasse 103, 8708 Männedorf, Switzerland).

### 2.3. Stopped Colorimetric Signal in Solution

**Figure 2:**
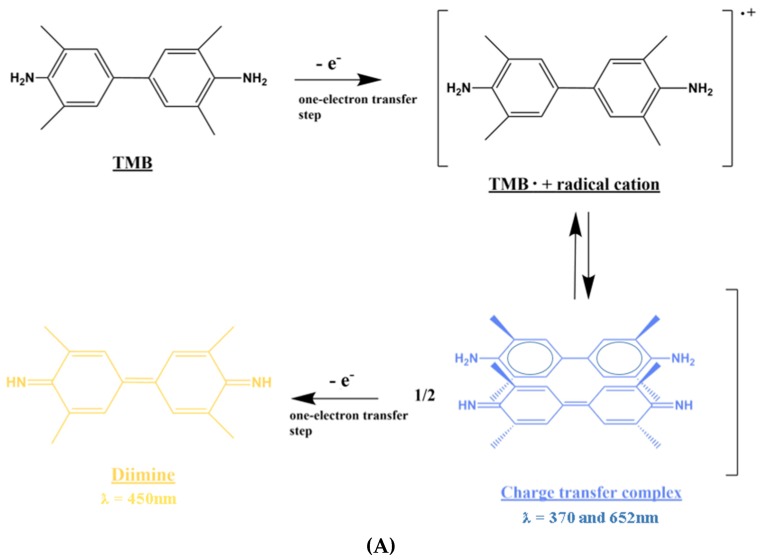

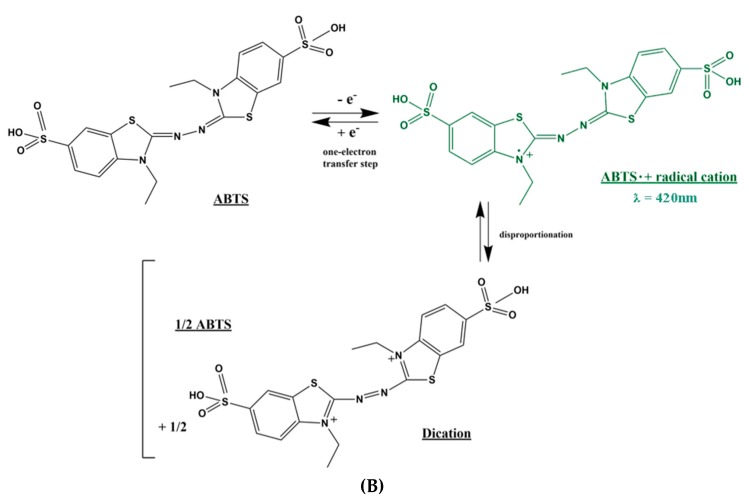
Colorimetric reaction of HRP with the Chromogenic Substrates TMB and ABTS. The colorimetric signal was generated based on the reaction of the enzyme horseradish peroxidase (HRP) with the chromogenic substrates (**A**) 3,3’,5,5’-tetramethylbenzidine (TMB) and (**B**) 2’-azinobis (3-ethylbenzothiazoline-6-sulfonic acid) (ABTS). Sulfuric acid and oxalic acid were used as the stopping solutions for TMB and ABTS respectively [26].

The stopped colorimetric signal was first tested in solution inside a 96-well plate. The two substrates were tested, TMB and ABTS, for the stopped colorimetric signal with HRP enzyme (Figure 2). In both cases, 50 ng/mL of HRP-antibody solution was used. In the case of TMB, three sulfuric acid concentrations (0.5, 1 and 2 M) were tested as stopping solutions, and the stopped colorimetric signal was measured at the wavelength of 450 nm. In the case of ABTS, three oxalic acid concentrations (0.3, 0.6 and 1 M) were tested as stopping solutions, and the stopped colorimetric signal was measured at 405 nm. Firstly, 100 µL of 50 ng/mL HRP-antibody, diluted in PBS buffer, was added to each well, together with 100 µL of substrate (either TMB or ABTS). After 8 min of reaction at RT and in dark conditions, 100 µL of stopping solution was added accordingly. The absorbance signal was then immediately measured using a TECAN reader. The stopped colorimetric signal was further tested with a range of HRP-antibody concentrations 0–75 ng/mL (0, 0.5, 1, 2.5, 10, 25, 50 and 75 ng/mL) for both substrates. In this case, the stopping solutions used for TMB and ABTS were kept at 0.5M sulfuric acid and 0.6M oxalic acid respectively.

### 2.4. Unstopped Colorimetric Signal on Membrane

Colorimetric signal detection was then carried out on membrane in order to compare unstopped and stopped colorimetric signals detection in paper diagnostics. Firstly, the unstopped colorimetric signal on membrane was examined for two HRP substrates, TMB and ABTS, and examined over a range of HRP-antibody dilutions. The 0.5 mg/mL HRP-antibody stock concentration was diluted in 0.05% (v/v) PBS-Tween20 buffer into the following dilutions: 1:4K; 1:6K; 1:8K; 1:10K; 1:12K; 1:14K; 1:16K and 1:18K. The substrate, TMB or ABTS, was first immobilized on a 6mm Ø cut cellulose absorption pad. Four different substrate volumes (10, 20, 30 and 40 µL) were then tested for each substrate. After placing the substrates on the absorption pads, the pads were dried at RT for 2 h. In order to test for unstopped colorimetric signal reaction on the membrane, the pads were then exposed to a 50 µL drop of diluted HRP-antibody accordingly. After 8 min of reaction, the pads were visually inspected and a picture was taken for further examination.

### 2.5. Stopped Colorimetric Signal on Membrane

Subsequently, stopped colorimetric signal detection on membrane for paper diagnostics was also examined. The stopped colorimetric signal on membrane was tested for the two substrates, TMB and ABTS, with 100 ng/mL HRP enzyme, and with or without a PVDF membrane as a separation layer. The detection of stopped colorimetric signal on membrane was examined using ‘Stack-Pad’ sensor layout, which consists of vertically stacked functional membranes (Figure 1) [21,22]. The three layers of PVDF membrane serve as a separation layer between the functionalized substrate and the stopping layers in the ‘Stack-Pad’. The sample is added from the bottom-up and as the layers are wetted, the sample moves upwards. In the top-most layer, a colorimetric signal was generated from the reaction between the substrate and the HRP-conjugated antibody. The ‘Stack-Pad’ test was constructed as follows: (from bottom up) 0.05% PBS-Tween20 immobilized on ‘sample layer’ (glass fiber membrane), 50 µL of 100 ng/mL HRP-antibody (diluted in 0.05% PBS-Tween20) immobilized on the ‘conjugation layer’ (polyester membrane), 1% (w/v) polyvinyl alcohol (PVA) immobilized on ‘buffer layer’ (polyester membrane), 50 µL substrate (TMB or ABTS) immobilized on the ‘substrate layer’ (polyester membrane), unmodified ‘separation layer’ (PVDF membrane) and 50 µL stopping solution (0.5M sulfuric acid for TMB and 0.6M oxalic acid for ABTS) immobilized on the ‘stopping layer’ (cellulose membrane). After the immobilization of the chemicals, all pads were dried for 1–2 h at RT. After 8 min of reaction, the pads were visually inspected and a picture was taken for further examination. The experiment was also conducted for the unstopped reaction, as a measure of control for the generation of an unstopped colorimetric signal with and without the use of the PVDF membrane as a separation layer. In addition, in order to examine the colorimetric signal stability over time, the signals were further examined in several time points.

### 2.6. Color Intensity Analysis

The images of the pads color intensity were firstly recorded in JPEG files. Then, they were analyzed using Image J software (National Institutes of Health and the Laboratory for Optical and Computational Instrumentation, University of Wisconsin, Madison, WI, USA). The colored images were firstly transformed to a 32-bit format and converted into grey contrast. Thereafter, the images were inverted for further analysis. Lastly, the average color intensities were recorded for each tested pad.

## 3. Results and Discussion

### 3.1. Stopped Colorimetric Signal in Solution

**Figure 3:**
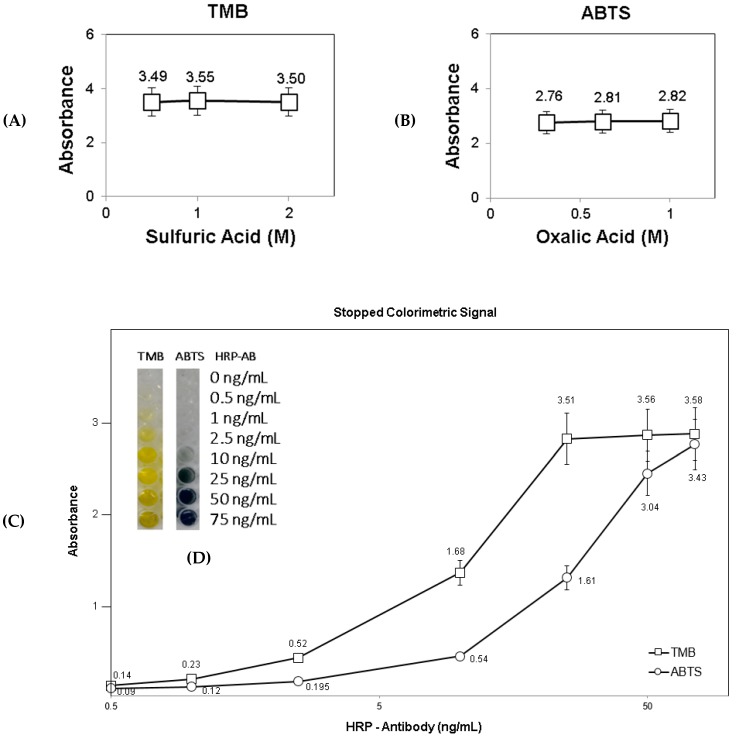
Stopped Colorimetric Signal in Solution. (**A**) Stopped colorimetric signal for TMB (450nm) with three sulfuric acid concentrations (0.5, 1 and 2 M) as a stopping solution, using 50 ng/mL HRP-antibody concentrations. (**B**) Stopped colorimetric signal measurement for ABTS (405nm) with three oxalic acid concentrations (0.3, 0.6 and 1 M) as a stopping solution, using 50 ng/mL HRP-antibody concentrations. (**C**) Stopped colorimetric signal of TMB (450nm; 0.5M sulfuric acid) vs. ABTS (405nm; 0.6M oxalic acid) with a range of HRP-antibody concentrations. TMB showed increased sensitivity, with higher absorbance signals for the same HRP-antibody concentrations. (**D**) Visual examination of the stopped colorimetric signal for TMB (450 nm) and ABTS (405 nm) with a range of HRP-antibody concentrations.

Firstly, the stopped colorimetric signal was compared in solution, and the results are presented in Figure 3. The two substrates, TMB and ABTS, were both exposed to the same HRP-antibody concentration of 50 ng/mL. For each substrate, three different concentrations of stopping solution were tested [27]. In the case of TMB, sulfuric acid solution was used as the stopping solution (0.5, 1 and 2 M) [28,29]. As shown in Figure 3A, in all three tested concentrations of stopping solution, the stopped signal measured was approximately the same (3.49, 3.55 and 3.50 absorbance). Similarly, for ABTS, as shown in Figure 3B, the three tested concentrations of oxalic acid stopping solution (0.3, 0.6 and 1 M) [30,31] showed similar stopped signal values (2.76, 2.81 and 2.82 absorbance) as well. Therefore, because the absorbance signal was similar for all the three tested concentrations of the stopping solutions and based on the reported previous studies, 1M sulfuric acid and 0.6M oxalic acid were selected as stopping solutions for TMB and ABTS accordingly. The stopped colorimetric signal was further tested with a range of HRP-antibody concentrations from 0–75 ng/mL (0, 0.5, 1, 2.5, 10, 25, 50 and 75 ng/mL) for both substrates (Figure 3C). The stopped signals for TMB and ABTS were tested with 0.5M sulfuric acid and 0.6M oxalic acid stopping solutions respectively. As observed in Figure 3C, TMB showed increased sensitivity in the stopped colorimetric signal in all of the tested HRP-antibody concentrations (0 ng/mL: 0.077 vs. 0.066; 0.5 ng/mL: 0.138 vs. 0.098; 1 ng/mL: 0.229 vs. 0.119; 2.5 ng/mL: 0.519 vs. 0.195; 10 ng/mL: 1.68 vs. 0.540; 25 ng/mL: 3.51 vs. 1.61; 50 ng/mL: 3.56 vs. 3.04 and 75 ng/mL: 3.58 vs. 3.43). The increased TMB sensitivity in the stopped colorimetric signal was mainly detected between 1–25 ng/mL HRP-antibody concentrations. The color gradient in the stopped colorimetric signal is also clearly visible for the range of HRP-antibody concentrations tested, as shown in Figure 3D. To conclude, a stopped colorimetric signal was detected in solution for both tested substrates, TMB and ABTS, with a clear color gradient observed for the range of HRP-antibody concentrations. It was also observed that measurements obtained from stopping the reaction were stable over the next several hours. On the contrary, in the unstopped signal detection, the reaction continues even during measurement, resulting in a less stable signal obtained. Stopping the reaction of colorimetric signal generation thus not only enhances the signal detection, but also stabilizes the detected signal, allowing a more accurate signal to be detected in comparison to unstopped colorimetric signal.

### 3.2. Unstopped Colorimetric Signal on Membrane

**Figure 4:**
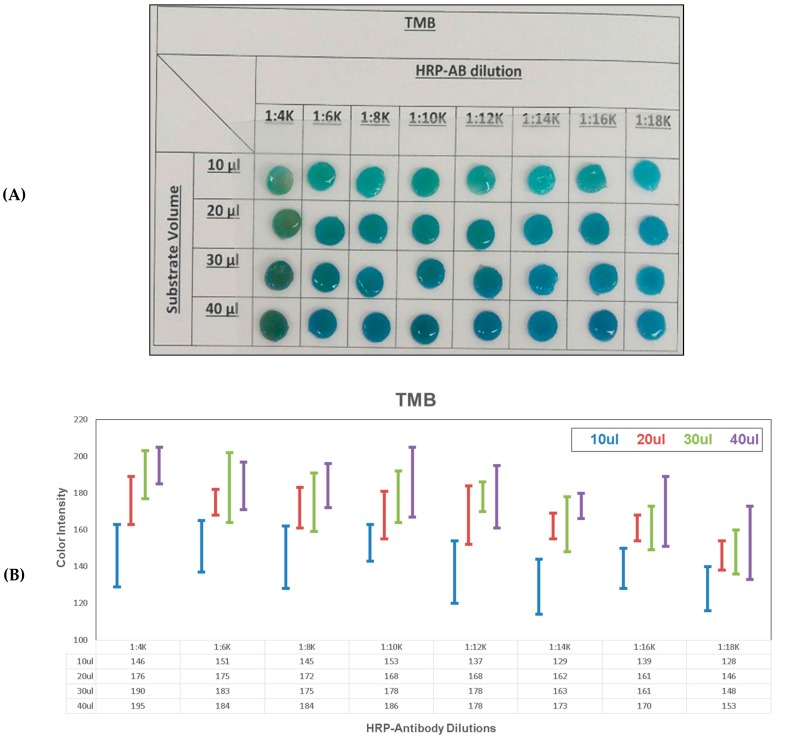
TMB unstopped colorimetric signal on membrane. Colorimetric signal on membrane with a range of HRP-antibody concentrations and a range of TMB substrate volumes: (**A**) picture and (**B**) color intensity analysis.

**Figure 5:**
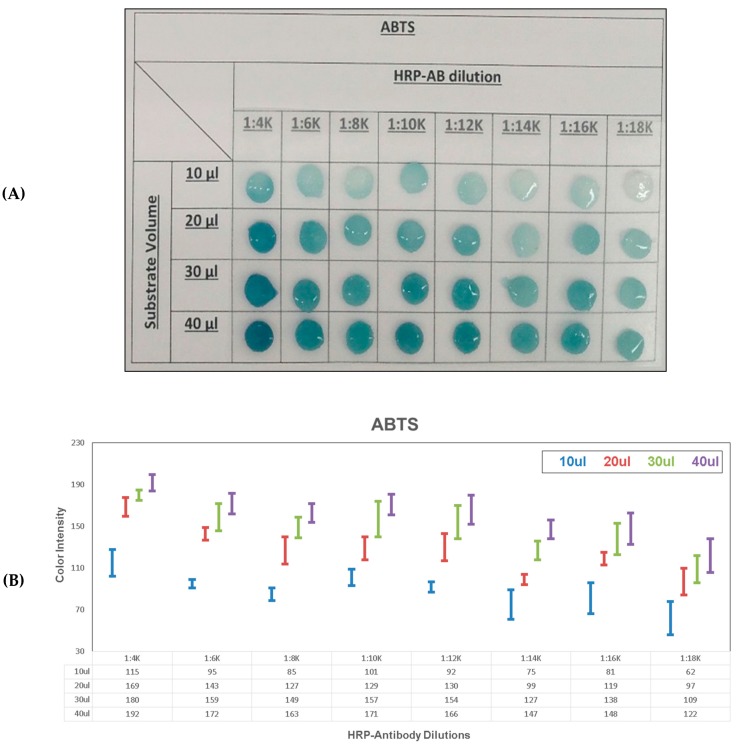
ABTS unstopped colorimetric signal on membrane. Colorimetric signal on membrane with a range of HRP-antibody concentrations and a range of ABTS substrate volumes: (**A**) picture and (**B**) color intensity analysis.

Colorimetric signal detection was then carried out on membrane, in order to compare unstopped and stopped colorimetric signals detection in paper diagnostics (Figure 4 and Figure 5, Table 1). The unstopped colorimetric signal on membrane was compared for the two HRP substrates, TMB and ABTS, and also examined with a range of HRP-antibody dilutions in 0.05% (v/v) PBS-Tween20 buffer (1:4K; 1:6K; 1:8K; 1:10K; 1:12K; 1:14K; 1:16K and 1:18K). Four different substrate volumes (10, 20, 30 and 40 µL) were also tested for each substrate. TMB (Figure 4) showed an overall higher sensitivity for the diluted HRP-antibody samples compared to ABTS (Figure 5). In the lowest substrate volume tested of 10 µL, ABTS did not show any unstopped colorimetric signal generated for the case of 1:18K HRP-antibody dilution. However, the unstopped colorimetric signal is clearly visible for 10 µL TMB with 1:18K HRP antibody. In the color intensity analysis (Figure 4B and Figure 5B), the results showed a clear gradient for the HRP-antibody dilutions (40 µL substrate: TMB: 1:4K (195) vs. 1:18K (153) and ABTS: 1:4K (192) vs. 1:18K (122)). As well as, a clear gradient for the substrate volume (1:4K HRP-antibody: TMB: 10 µL (146) vs. 40 µL (195) and ABTS: 10 µL (115) vs. 40 µL (192)). In general, a clear gradient is visible for both substrates tested, over the range of the different tested substrate volumes and HRP-antibody dilutions. However, as the unstopped colorimetric signal in TMB was generally stronger and produced more intense coloration in all of the tested HRP-antibody dilutions, the unstopped colorimetric signal gradient was thus more easily observed in the case of ABTS. To conclude, the unstopped colorimetric signal on membrane can be clearly observed for both TMB and ABTS, for the tested HRP-antibody concentrations. A colorimetric signal can be generated on membrane using both TMB and ABTS substrates with HRP conjugated antibodies.

### 3.3. Stopped Colorimetric Signal on Membrane

**Figure 6:**
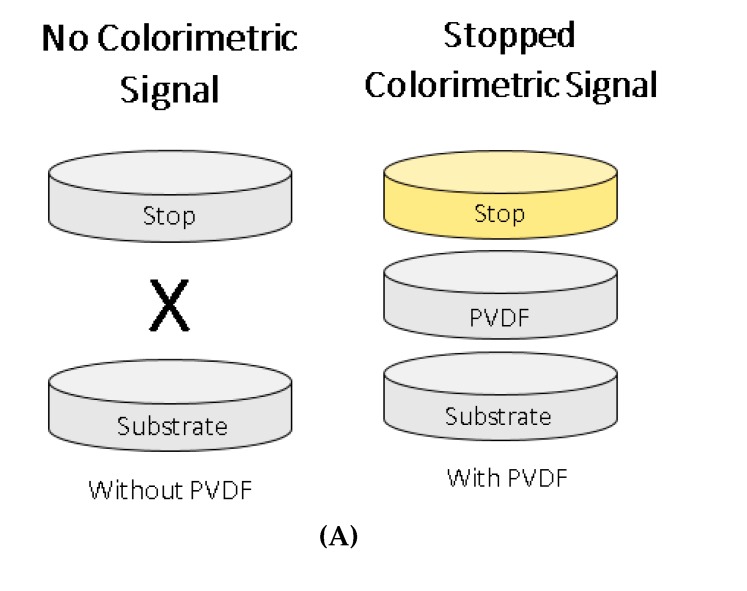

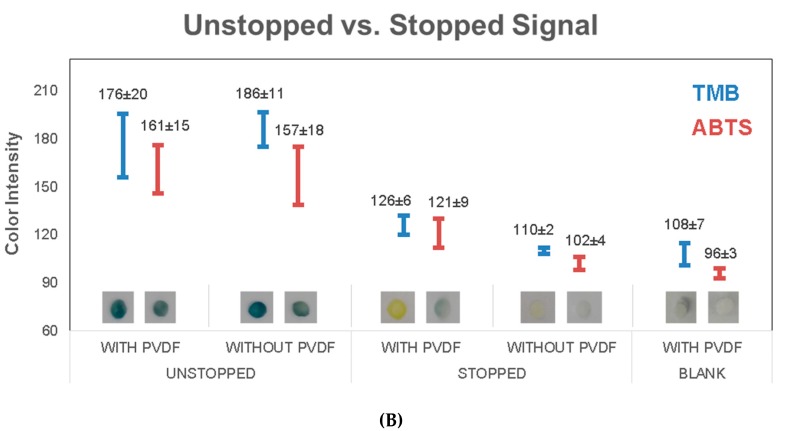
Stopped colorimetric signal on membrane. (**A**) The stopped colorimetric signal was examined with and without three layers of polyvinylidene difluoride (PVDF) membrane as a separation layer between the functionalized substrate and the stopping layers. (**B**) Two substrates were compared: 3,3’,5,5’-tetramethylbenzidine (TMB) vs. 2’-azinobis (3-ethylbenzothiazoline-6-sulfonic acid) (ABTS), for unstopped vs. stopped colorimetric signal comparison on membrane, using 100 ng/mL HRP-antibody.

Stopped colorimetric signal was detected on membrane, using ‘Stack-Pad’ sensor layout [21,22] (Figure 1), which consists of vertically stacked functional membranes (Figure 6). The stopped colorimetric signal on membrane was tested for the two substrates, TMB and ABTS, with 100 ng/mL HRP enzyme, and with or without a PVDF separation layer. The comparison between the unstopped vs. stopped colorimetric signal was also conducted. Firstly, the unstopped colorimetric signal was detected for both cases of TMB and ABTS, with and without the use of PVDF [24] as a separation layer. The unstopped reaction was conducted as a measure of control for the generation of an unstopped colorimetric signal with and without the use of PVDF as a separation layer. The unstopped signals obtained for both substrates, TMB and ABTS, are visibly similar in the cases of with and without PVDF membrane (Figure 6). This suggests that the use of PVDF membrane as a separation layer does not interfere with the generation of a colorimetric signal. For the generation of stopped colorimetric signal on membrane, a clear, yellow stopped colorimetric signal was visible on the membrane when TMB substrate was used in the presence of a PVDF layer. However, in the case of ABTS substrate, the colorimetric signal was less visible. When the stopped colorimetric signals obtained with PVDF separation layer were compared to the signals obtained without PVDF, it was observed that the stopped colorimetric signals were not visible without PVDF. These results strengthen the importance of using PVDF membrane as a separation layer between the substrate and the stopping solution layers, for the generation of a stopped colorimetric signal. This can be explained by the hydrophobic nature of the PVDF membrane, which slows the flow of sample between the substrate and the stopping solution layers, giving rise to a buffer layer for the generation of a colorimetric signal [24]. The results of the color intensity analysis also support these findings (Figure 6). The blank control with PVDF showed similar signal level (TMB: 108 and ABTS: 96) as the stopped test without PVDF (TMB: 110 and ABTS: 102). Meaning, that without PVDF membrane as separation layer, the stopped signal was not visually and quantitatively detected. Moreover, when comparing the stopped signal with and without PVDF membrane, the stopped signal detected with PVDF showed an increased value (TMB: 126 and ABTS: 121). In addition, the unstopped signal for both with (TMB: 176 and ABTS: 161) and without (TMB: 186 and ABTS: 157) PVDF membrane did show an elevated value, this is expected since the colorimetric reaction is stopped. However, looking closer at the standard deviation values, the stopped signal is more accurate with lower standard deviation values compared to the unstopped signal. These quantitative signal value also provide evidence to support the claim that the addition of PVDF membrane as separation layer does not interfere to the generation of the colorimetric signal (TMB: with: 176 vs. without: 186 and ABTS: with: 161 vs. without: 157). To conclude, the stopped colorimetric signal was clearly observed for TMB but less observed for ABTS. Moreover, the generation of stopped colorimetric signal was dependent on the presence of PVDF membrane as separation layer between the two functionalized layers of substrate (TMB) and stop solution (sulfuric acid).

### 3.4. Colorimetric Signal Stability Over Time

**Figure 7:**
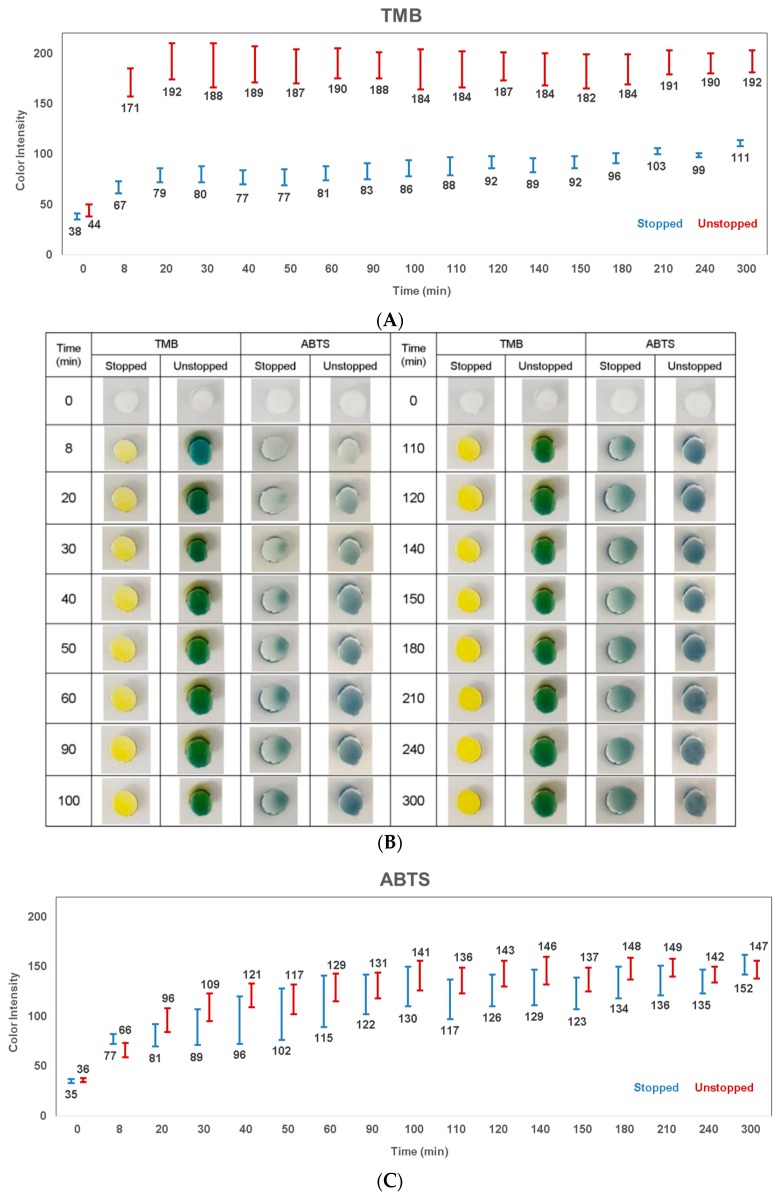
Stopped vs. unstopped colorimetric signal stability over time. Two substrates were compared: 3,3’,5,5’-tetramethylbenzidine (TMB) vs. 2’-azinobis (3-ethylbenzothiazoline-6-sulfonic acid) (ABTS) for unstopped vs. stopped colorimetric signal comparison on membrane. (**A**) TMB; (**B**) picture; (**C**) ABTS.

The colorimetric signal stability was also examined further over several time points (Table 2 and Figure 7). The stability of both stopped and unstopped colorimetric signals on membrane were compared, with three layers of PVDF membrane as a separation layer between the substrate and the stopping solution layers. In the case of TMB (Figure 7A), it is clear that the unstopped signal values (171–192) were higher than the stopped signal values (67–111). However, looking closer at the standard deviation values, it is possible to determine that the stopped colorimetric signal demonstrated a more stable and accurate signal detection with lower standard deviation values. On the contrary, in the case of ABTS (Figure 7C), both unstopped (66–149) and stopped (77–152) signals were in a similar range. This might indicate that the ABTS colorimetric reaction was not stopped. However, from both the image (Figure 7B) and color intensity analysis (Figure 7C), it is possible to still determine that there was a slight difference between the unstopped and stopped colorimetric signal. A difference is already visible after the 20 min time point (stopped: 81 ± 11 vs. unstopped: 96 ± 12), and can be identified until the 100 min time point (stopped: 130 ± 20 vs. unstopped: 141 ± 15). The ABTS stopped colorimetric reaction developed slightly slower than the unstopped reaction. To conclude, the stopped colorimetric signal was more stable and accurate over time for TMB but less observed for ABTS.

## 4. Conclusions

Firstly, the stopped colorimetric signal was compared in solution. Two substrates, TMB vs. ABTS, were exposed to the HRP-antibody. For each substrate, three different concentrations of stopping solutions were tested. In the case of TMB, sulfuric acid solution was used as the stopping solution (0.5, 1 and 2 M), and the stopped signal obtained in each case was approximately the same (3.49, 3.55 and 3.50 absorbance). Similarly, in the case of ABTS, oxalic acid (0.3, 0.6 and 1 M) was used as the stopping solution and the stopped signals obtained were approximately the same (2.76, 2.81 and 2.82 absorbance). The stopped colorimetric signal was then further tested with a range of HRP-antibody concentrations. TMB showed increased sensitivity in the stopped colorimetric signal in all the tested HRP-antibody concentrations. In order to compare unstopped and stopped colorimetric signal detection in paper diagnostics, the unstopped colorimetric signal on membrane was also examined, with a range of HRP-antibody dilutions. TMB showed increased sensitivity for the diluted HRP-antibody samples as compared to ABTS. In general, a clear gradient was visible for both substrates tested. Lastly, stopped colorimetric signal was detected on membrane, using ‘Stack-Pad’ sensor layout [21,22], which consists of vertically stacked functional membranes. The unstopped reaction was also conducted, as a measure of control for the generation of an unstopped colorimetric signal with and without the use of the PVDF separation layer. In the case of TMB substrate, a clear, yellow stopped colorimetric signal was visible on the membrane. However, in the case of ABTS substrate, colorimetric signal was not visible. Moreover, when comparing between the stopped colorimetric signals obtained with PVDF as separation layer and those without, it was concluded that the stopped colorimetric signal was not visible without PVDF. To conclude, the stopped colorimetric signal was clearly observed for TMB but not for ABTS. Moreover, the generation of stopped colorimetric signal was dependent on the presence of PVDF membrane as a separation layer between the two functionalized layers of substrate (TMB) and stop solution (sulfuric acid). In addition, the stopped colorimetric signal was more stable and accurate over time for TMB but less observed for ABTS. This study concludes that stopped colorimetric signals can be generated in paper-based biosensors for enhanced and accurate signal detection.

## Figures and Tables

**Table 1:** Color intensity analysis for TMB and ABTS unstopped colorimetric signal on membrane.

Color Intensity									
TMB									
	HRP-Antibody Dilution								
		1:4K	1:6K	1:8K	1:10K	1:12K	1:14K	1:16K	1:18K
TMB Volume	10 µL	146 ± 17	151 ± 14	145 ± 17	153 ± 10	137 ± 17	129 ± 15	139 ± 11	128 ± 12
	20 µL	176 ± 13	175 ± 7	172 ± 11	168 ± 13	168 ± 16	162 ± 7	161 ± 7	146 ± 8
	30 µL	190 ± 13	183 ± 19	175 ± 16	178 ± 14	178 ± 8	163 ± 15	161 ± 12	148 ± 12
	40 µL	195 ± 10	184 ± 13	184 ± 12	186 ± 19	178 ± 17	173 ± 7	170 ± 19	153 ± 20
ABTS									
	HRP-Antibody Dilution								
		1:4K	1:6K	1:8K	1:10K	1:12K	1:14K	1:16K	1:18K
ABTS Volume	10 µL	115 ± 13	95 ± 4	85 ± 6	101 ± 8	92 ± 5	75 ± 14	81 ± 15	62 ± 16
	20 µL	169 ± 9	143 ± 6	127 ± 13	129 ± 11	130 ± 13	99 ± 5	119 ± 6	97 ± 13
	30 µL	180 ± 5	159 ± 13	149 ± 10	157 ± 17	154 ± 16	127 ± 9	138 ± 15	109 ± 13
	40 µL	192 ± 8	172 ± 10	163 ± 9	171 ± 10	166 ± 14	147 ± 9	148 ± 15	122 ± 16

**Table 2:** Color intensity analysis for stopped vs. unstopped colorimetric signal stability over time.

Color Intensity				
Time (min)	TMB		ABTS	
	Stopped	Unstopped	Stopped	Unstopped
0	38 ± 3	44 ± 6	35 ± 2	36 ± 2
8	67 ± 6	171 ± 14	77 ± 5	66 ± 7
20	79 ± 7	192 ± 18	81 ± 11	96 ± 12
30	80 ± 8	188 ± 22	89 ± 18	109 ± 14
40	77 ± 7	189 ± 18	96 ± 24	121 ± 12
50	77 ± 8	187 ± 17	102 ± 26	117 ± 15
60	81 ± 7	190 ± 15	115 ± 26	129 ± 14
90	83 ± 8	188 ± 13	122 ± 20	131 ± 13
100	86 ± 8	184 ± 20	130 ± 20	141 ± 15
110	88 ± 9	184 ± 18	117 ± 20	136 ± 13
120	92 ± 6	187 ± 14	126 ± 16	143 ± 13
140	89 ± 7	184 ± 16	129 ± 18	146 ± 14
150	92 ± 6	182 ± 17	123 ± 16	137 ± 12
180	96 ± 5	184 ± 15	134 ± 16	148 ± 11
210	103 ± 3	191 ± 12	136 ± 15	149 ± 9
240	99 ± 2	190 ± 10	135 ± 12	142 ± 8
300	111 ± 3	192 ± 11	152 ± 10	147 ± 9
